# Influence of Probe Flexibility and Gelatin Embedding on Neuronal Density and Glial Responses to Brain Implants

**DOI:** 10.1371/journal.pone.0119340

**Published:** 2015-03-19

**Authors:** Per Köhler, Anette Wolff, Fredrik Ejserholm, Lars Wallman, Jens Schouenborg, Cecilia E. Linsmeier

**Affiliations:** 1 Neuronano Research Center (NRC), Department of Experimental Medical Science, Lund University, Lund, Sweden; 2 Department of Measurement Technology and Industrial Electrical Engineering, Electrical Measurements, The Faculty of Engineering at Lund University, Lund, Sweden; National Institutes of Health, UNITED STATES

## Abstract

To develop long-term high quality communication between brain and computer, a key issue is how to reduce the adverse foreign body responses. Here, the impact of probe flexibility and gelatine embedding on long-term (6w) tissue responses, was analyzed. Probes of same polymer material, size and shape, flexible mainly in one direction, were implanted in rat cerebral cortex (n_implants_ = 3 x 8) in two orientations with respect to the major movement direction of the brain relative to the skull: parallel to (flex mode) or transverse to (rigid mode). Flex mode implants were either embedded in gelatin or non-embedded. Neurons, activated microglia and astrocytes were visualized using immunohistochemistry. The astrocytic reactivity, but not microglial response, was significantly lower to probes implanted in flex mode as compared to rigid mode. The microglial response, but not astrocytic reactivity, was significantly smaller to gelatin embedded probes (flex mode) than non-embedded. Interestingly, the neuronal density was preserved in the inner zone surrounding gelatin embedded probes. This contrasts to the common reports of reduced neuronal density close to implanted probes. In conclusion, sheer stress appears to be an important factor for astrocytic reactivity to implanted probes. Moreover, gelatin embedding can improve the neuronal density and reduce the microglial response close to the probe.

## Introduction

Biocompatible brain computer interfaces that allow long-term recordings of neural activity in the conscious individual have great potential in neuroscience [1]. Still, however, the ability to form stable electrical contacts with neurons in the central nervous system over long periods of time is limited [2]. Usually, the recording quality deteriorates over time, at least partly a result of glial scarring encapsulating the implanted electrodes [3,4,5]. It is therefore of importance to elucidate the key mechanisms that provoke glial proliferation and thus the glial scaring around the implant.

A common feature of state of art neural interfaces is that they are relatively rigid to enable implantation [6] and, via electrical connectors, tethered to the skull. Since the brain is floating in the skull, it has been suggested, that part of the probe-tissue reactions is due to microforces, which result from micromotions [7]. In a combined simulation and histological study, Subbaroyan and Kipke [8] simulated shear stress along the axis of an implanted probe tethered to the skull, resulting from the propagation of one dimensional micromotion in a finite element model. In the following histopathological setup, tissue reactions were found to match the micromotions modelled.

Multiphoton imaging of brain micromotions in head-restrained mice identify the principal axis of movement to be cephalocaudal [9], which is also seen in human MR imaging studies [10]. This is in line with previous results from our laboratory [11], in which rigid cylindrical probes implanted in rat cortex cerebri and tethered to the skull caused oval scarring, elongated cephalocaudally, indicating that this is the principal direction of brain movement relative to the skull of freely moving rodents.

Moreover, implanted untethered probes with a similar specific weight as the surrounding tissue elicit less chronic tissue response than heavier untethered implants [12,13]. It has also been suggested that flexible electrodes, that can follow tissue movements, are more tissue friendly than rigid electrodes but there is no conclusive support for this notion, as the flexible electrodes tested so far also differ in size or in materials [14,15]. Therefore, the initial part of the present study aims at confirming this notion. To avoid confounding factors arising from differences in probe design or materials [16], we chose to use *one* polymer probe, which differ greatly (by a factor of 8000) in flexibility with respect to direction and have made use of the fact that the dominant brain movement inside the skull occurs along the cephalocaudal axis during daily activities.[9,10] We then compared the long-term tissue responses (at six weeks) to the probe implanted in rat cerebral cortex in two different orientations: i.e. with its flexible direction parallel (flex mode) to or transverse (rigid mode) to the major direction of the movements of the brain relative to the skull.

Note that the probe used here was specifically designed to provide information on the impact of probe flexibility and gelatine embedding on tissue responses and not as an actual electrode. However, evidence that similar flat and flexible electrode arrays can indeed function normally in *in vivo* has been presented previously [17].

Assuming an important impact of probe flexibility could be confirmed, and that we in a previous study found that embedding a probe in gelatin reduces the astrocytic and microglial response [18], the main aim of the present study was to clarify if the glial response to a flexible probe can be further reduced by embedding the probe in gelatin.

## Materials and Methods

All animal-related procedures were conducted in accordance with local and international ethical guidelines, with the specific permission of the Lund and Malmö Ethical Board, diary number M258–11. All implantations (n_implantations_ = 24) were made in female Sprague-Dawley rats (n_rats_ = 20, Taconic, Denmark), weighing 200–250 g. Animals were anaesthetized using intraperitoneal (i.p.) injections of fentanyl (0.3 mg/kg body weight (b.w.)) and Domitor vet (medetomidin hydrochloride, 0.3 mg/kg b.w.). After six weeks, the animals were anaesthetized with an overdose of pentobarbital (i.p) and transcardially perfused with 150–200 ml ice-cold 0.1 M phosphate buffer (PB), followed by 4% paraformaldehyde (PFA) in 0.1 M PB. One animal was excluded for meeting ethical guideline exclusion criteria after suffering a soft tissue infection following surgery. Animals were kept in a 12 h light/dark cycle with access to food and water *ad libitum*.

### Probe fabrication

Single shank probes were manufactured in our in-house clean room facilities, as described below. A 3” silicon wafer was wet oxidized at 1200°C (6 h), resulting in an oxide thickness of about 1.2 μm. The resulting silicon oxide layer acts as a sacrificial layer in the end of the fabrication process. After oxidation the wafer was cleaned and activated using oxygen plasma (4 min).

A photoresist, SU-8 2005 (MicroChem), was spin coated onto the wafer, yielding a layer thickness of roughly seven μm. The wafer was subsequently soft baked (90°C, 20 min). The pattern was then defined by exposing the wafer to the desired pattern using a MA4 Karl-Suss mask aligner (12 s). The wafer was post exposure baked (90°C, 30 min), beginning and ending at room temperature.

To develop the pattern, the wafer was put into mr-Dev 600 developer (1 min, MicroChem), rinsed using isopropanol (VWR) and then blown dry using gaseous nitrogen. To ensure that all SU-8 was cross-linked, the wafer was hard baked (210°C, 3.5 h). As a final step, the wafer was immersed in HF:H_2_0 1:5 (MilliQ) for about two days in order to etch the silicon dioxide layer and release the final structures. To ensure that no HF was left, the HF:H_2_0 was replaced with water four times.

**Fig 1:**
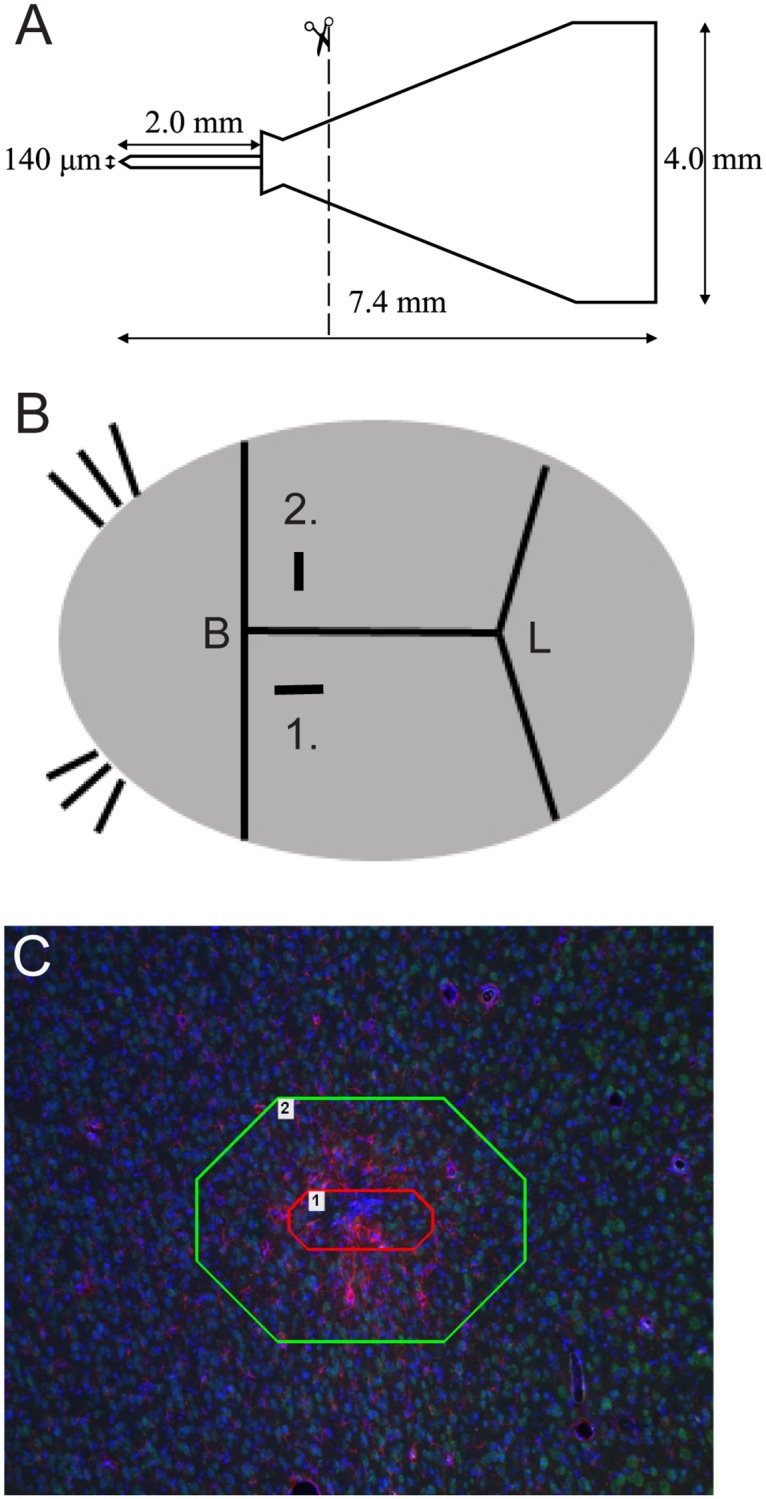
Visualization of the probe used for implantation, implantation modes and regions of interest for image analysis. A) Schematic overview of the probe. Images are not to scale. B) Shanks were inserted 1 mm caudal and 2.3 mm lateral to Bregma (B, Bregma), either sagittal (rigid mode, 1) or coronal (flex mode, 2). (L, Lambda). C) Two regions of interests (ROIs) were analyzed in NIS Elements 3.1 software (Nikon, Japan), measuring 0–50 μm (inner ROI, marked with a red line) and 50–200 μm (outer ROI, marked with a green line) from the estimated border of the hole left by the implant.

The final probe (Fig. 1A) was two mm in total length, with a width of 140 μm at the base with a tapered tip, and a thickness of roughly seven μm. Since flexural rigidity scales cubically, the probe was 20^3^ = 8000 times more flexible in the seven μm thickness direction, as compared to the 140 μm width direction.

To facilitate handling and implantation, the probes were mounted on stainless steel guiding wires (50 μm in diameter), approximately three mm long, using a sucrose solution as an adhesive. Furthermore, the probes that were implanted in the flex mode were either embedded in a 5–10 μm thick gelatin matrix or non-embedded, as previously described (17).

### Experimental groups

Animals were divided into three experimental groups: rigid (flat probe side implanted in the sagittal plane (Fig. 1B)) mode, flex (flat probe side implanted in the coronal plane (Fig. 1B)) mode and gelatin embedded probes implanted in flex mode. Animals were implanted unilateral, in groups of eight, except the rigid mode group that were implanted bilateral in four animals, as we in a parallel study [19] found that there is no significant interaction between multiple implants.

### Surgical procedures

Animals were anaesthetized using intraperitoneal (i.p.) injections of fentanyl (0.3 mg/kg body weight (b.w.)) and Domitor vet (medetomidin hydrochloride, 0.3 mg/kg b.w.) and placed in a stereotactic frame for surgery. A rostrocaudal incision in the skin was placed along the central suture of the skull to expose Bregma. A round opening approximately two mm across was made 1.0 mm caudal to Bregma and 2.3 mm lateral to the mid line, and the dura mater was cut open using forceps and a syringe. The polymer probes, mounted on stainless steel guiding wires, were then implanted into the cortex to a depth of 2.0 mm using a micromanipulator. After rinsing the surface of the cortex with physiological saline to dissolve the sucrose, the guides were retracted and removed and the opening in the skull filled using FujiChem silastic, tethering the implant to the skull. Afterwards the wound was closed using surgical staples and the animals received subcutaneous injections of an antidote to the anesthesia (Antisedan, atipamezole hydrochloride, 0.5 mg/kg b.w.) as well as Temgesic (buprenorphine, 50 μg/kg b.w.) to reduce postoperative pain.

### Tissue fixation and sectioning

After six weeks, the animals were anaesthetized with an overdose of pentobarbital (i.p) and transcardially perfused with 150–200 ml ice-cold 0.1 M phosphate buffer (PB), followed by 4% paraformaldehyde (PFA) in 0.1 M PB. Brains were dissected using standard surgical tools and probe shanks were removed by pulling the silastic to which it was attached. In a few cases, probe shanks broke upon removal with fragments remaining in the tissue. No difference was later detected between these animals and animals where probes were removed upon dissection.

The brains were postfixed in 4% PFA overnight and then soaked in 30% sucrose for at least 24 hours for cryopreservation. They were then serially sectioned in the horizontal plane at 30 μm, using a cryostat (Microm HM560). Sections were kept in antifreeze in a free-floating manner.

### Immunohistochemistry

The astrocyte proliferation, the recruitment of microglial cells and the neuronal cell bodies were evaluated using free-floating immunohistochemical techniques. Hence, following rinses in phosphate buffered saline (PBS, pH 7.4), and preincubation in a mixture of 5% normal serum and 0.25% Triton X-100 (Sigma, Germany) in PBS, the sections were reacted with primary antibodies overnight at RT. The primary antibodies used were rabbit polyclonal antibodies recognizing Glial Fibrillary Acidic Protein (GFAP, an astrocytic cytoskeleton protein 1:5000, Dako, Denmark), and mouse monoclonal antibodies recognizing either CD68/ED1 (expressed by activated microglia/macrophages, 1:100, Serotec, USA) or NeuN (expressed on neuronal nuclei 1:100, Millipore, USA). After repeated rinses in PBS, the sections were further incubated in Alexa488-conjugated antibodies for mouse IgG and Alexa594-conjugated antibodies for rabbit IgG (1:500, Invitrogen, USA) (2 h, dark, RT) and rinsed in PBS. Sections were then mounted on Superfrost Ultra Plus glass slides and cover slipped using Vectashield Hardset mounting media with DAPI (cell nuclei marker, Vector, USA). Antibody staining with a specific antibody was performed in the same batches and thus handled alike to minimize staining variance.

### Image processing

All histological fluorescence image analysis were obtained using a DS-Ri1 Digital camera (Nikon Instruments, Japan) mounted on a Nikon Eclipse 80i microscope with a 10x objective (Nikon Instruments, Japan). For all images of a certain marker, identical exposure times, gains and filters were used. The images were acquired and analysed using the NIS-Elements BR software 3.2 (NIS-elements, Nikon Instruments, Japan). Due to difficulties in delineating individual cells when using some of our antibodies (ED1 and GFAP), two specific evaluation methods were used for the different immunostainings. Thus, for the NeuN and DAPI stainings, manual counts was performed, and for the glial markers GFAP and ED1, fluorescence intensity measurements was used as described previously [11]. Analyzed sections were chosen 920–1100 μm from the surface of the cortex, corresponding to rat cortical layer 4 as identified by the Paxinos and Watson rat brain atlas (6^th^ edition, 2007, Elsevier). Regions of interest (ROIs), as shown in Fig. 1C, were set at 0–50 μm (inner ROI) and 50–200 μm (outer ROI) from the rim of the artifact caused by the implants, if indeed a hole was detected. If no hole was evident, a line with the length of 140 μm was drawn, which corresponds to the width of the implant, and the distances were then set from the drawn line. The fluorescence intensity measurements were made by measuring the proportion of immunoreactive area (for ED1 and GFAP) in the total screened area for each marker in all experimental groups. Due to variability in the binding specificity of ED1 and GFAP to their respective antigen, thresholds were set for each individual image at a fixed ratio of the mean background intensity for each marker. The thresholds were set to ensure that only positively stained antigens were quantified, whereas the nonspecific background staining was not. The threshold for the signal to background ratio was set to 4.5 for ED1 and to 6.0 for GFAP immunofluorescence, respectively. The fraction of the area above this threshold in each ROI was quantified. For NeuN and DAPI, manually counts were performed in the two respective ROIs for all experimental groups.

Furthermore, to analyse neuronal cell survival, matched NeuN-positive cells were also counted in identical ROIs placed in naïve areas of the cortex. Sections from the midpart of the implant were analysed. Descriptive images were obtained using a 2-photon Zeiss Laser Scanning Microscope 710.

### Statistical analysis

Kruskal Wallis with Dunn’s multiple comparison test was used to compare the experimental groups, as well as neuronal survival fractions. Wilcoxon matched-pairs signed rank test was used when paired comparisons of two experimental groups was needed.

P-values < 0.05 were considered significant. All values are presented as median values with indication of the 25th to 75th percentiles. All analyses were performed using the GraphPad Prism 5.02 software (GraphPad Software Inc., USA).

## Results

Explanted brains were stained and analyzed for GFAP in order to assess the degree of reactive astrocytosis, for ED1 to assess the acute inflammatory response to the implanted probes and NeuN to assess neuronal survival in close vicinity to the implanted probe. Probes, implanted in the rigid vs. the flex mode, were compared to confirm the importance of probe flexibility on tissue responses. Results from gelatin embedded vs. non-embedded flex mode probes were then compared in order to investigate the effect of gelatin embedding. For all groups, tissue reactions elicited in the outer ROIs were too small to be detectable.

### General tissue reactions

**Fig 2:**
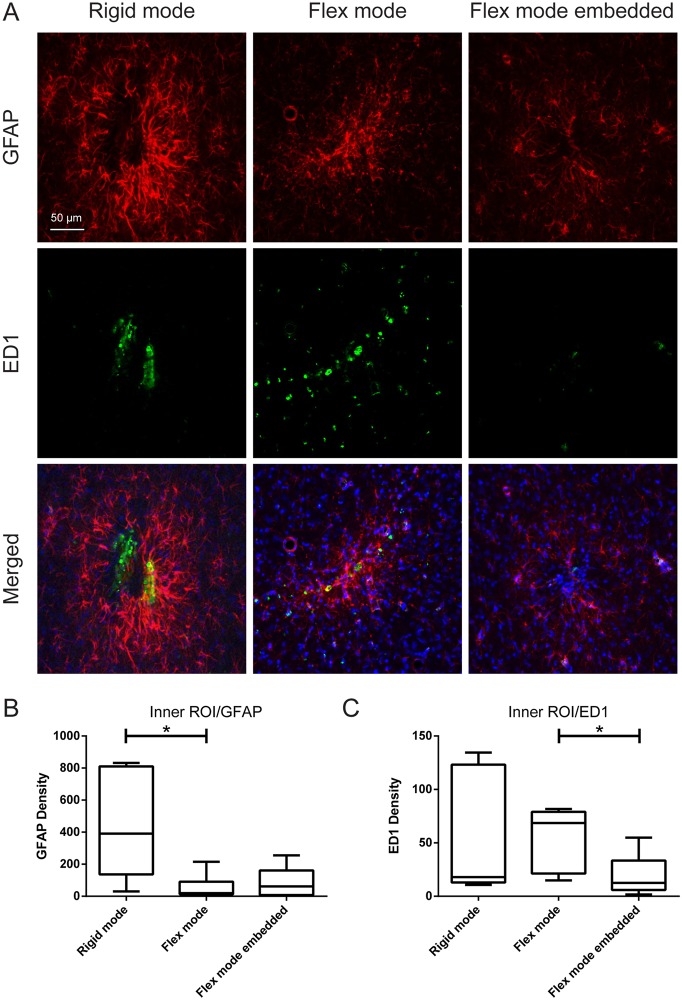
Implantation mode and embedding dependent glial reactions. A) Representative images of rigid, flex and flex embedded mode implantations after 6 weeks, showing mode dependent alterations in astrocytosis, as shown in red (GFAP), microglia responses, as shown in green (ED1) and merged (DAPI in blue (total cell nuclei)). Scale bar: 50 μm. Cephalocaudal direction corresponds to top-to-bottom in image. B and C) Quantification of the GFAP and ED1 densities, respectively, that surrounds the different implantation sites in the inner ROI (0–50 μm). The box corresponds to the 25th and 75th percentiles, the median value is indicated by the horizontal line within each box, and the whiskers show the minimum and maximum values. The horizontal lines indicate statistical differences.

**Fig 3:**
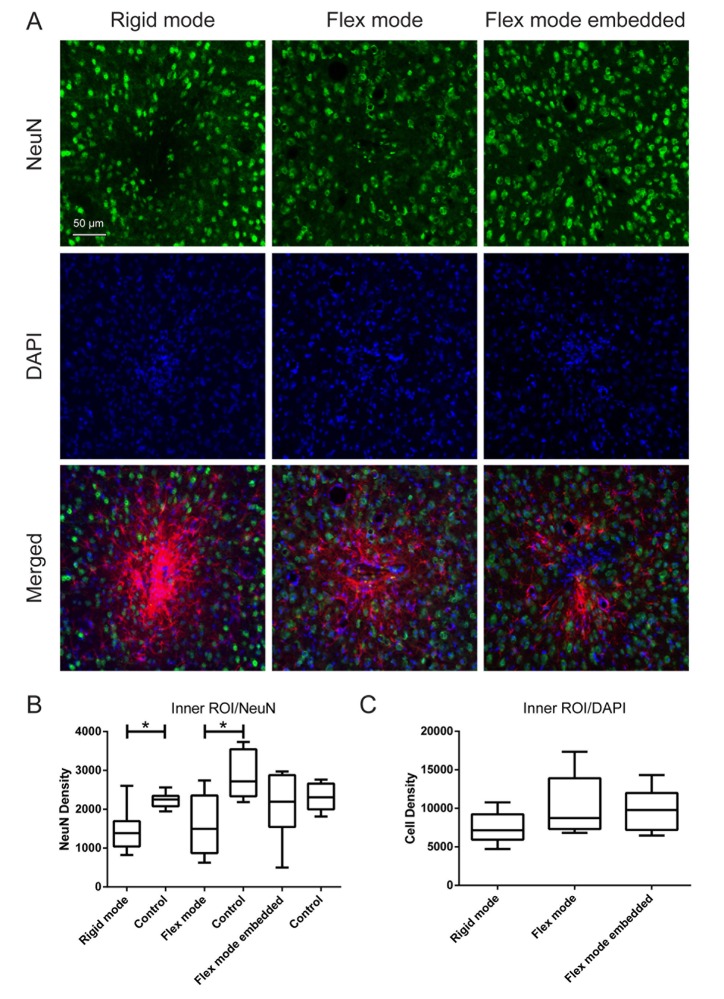
Effects on neuronal density and morphology. A) Representative images of rigid, flex and flex embedded mode implantations after 6 weeks, showing mode dependent alterations neuronal densities, as shown in green (NeuN), total cell nuclei (DAPI) in blue and merged (GFAP in red depicting the implantation scar). Scale bar: 50 μm. B and C) Quantification of the NeuN and DAPI densities, respectively, that surrounds the different implantation sites at the inner ROI (0–50 μm). Note that in B) the NeuN densities are compared to paired naïve areas (controls). The box corresponds to the 25th and 75th percentiles, the median value is indicated by the horizontal line within each box, and the whiskers show the minimum and maximum values. The horizontal lines indicate statistical differences.

Generally, we found that significant astrocytic reactions as well as significant microglia responses were restricted to the inner ROIs (Fig. 2A) of the implanted probes. However, no significant effect on the DAPI cell count between experimental groups, were found for either of the two ROIs (Fig. 3C for inner ROI results).

### Mode dependent glial reactions

Implantation in the flex mode, as compared to the rigid mode, was found to significantly decrease GFAP density in the inner (p = 0.0117) (Fig. 2B) but not the outer ROI (data not shown). By contrast, no significant difference in ED1 density between flexible and rigid modes was found in any of the ROIs (for inner ROI, Fig. 2C)

### Gelatin-embedding

Embedding the implants with gelatin, produced a statistically significant (p = 0.0378) reduction in microglial (ED1) density as compared to the non-embedded experimental group (flex mode embedded vs. non-embedded) (Fig. 2C). However, no differences were observed in astrocytic density between embedded vs. non-embedded probes (Fig. 2B).

### Neuronal survival after probe implantation

The neuronal survival in the inner and outer ROIs was compared to NeuN positive cells in naïve tissue, respectively, in all three experimental groups. Fig. 3A-B shows the neuronal density in the inner ROI. As can be seen, a significant decrease of neuronal density was found around probes in both the rigid mode, as well as around probes in the flex mode as compared to its respective controls. Notably, neuronal density was not reduced in tissue surrounding gelatin embedded probes. No differences were observed in neuronal density in the outer ROI (not shown), when compared to control.

Furthermore, neuronal nuclei in the inner ROI appeared more flattened in animals with probes implanted in the rigid mode as compared to in the flex mode. The effect on neuronal morphology was most marked around the edges of the implants.

## Discussion

We here show that the long-term proliferation of astrocytes, but not microglia recruitment, around an implanted probe tethered to the skull depends on the flexibility of the probe relative to the dominant movement of the brain inside the skull. We also provide evidence that gelatin embedding reduce the microglia responses to the implanted probe (flex mode), while having little additional effect on the long-term astrocytic reactions. It is also noticeable that there was no tendency for a reduction in neuronal density adjacent to the gelatin embedded probe, while non-embedded probes exhibited a significantly reduced number of neurons in the adjacent tissue as reported previously for most types of implanted probes [20,21]

### Effects of probe flexibility

There is a common notion within the field of brain computer interfacing that flexible implants is more tissue friendly than rigid ones, since thin and therefore more flexible implants tend to elicit less tissue reactions [11]. However, the impact of the flexibility per se of the probe on glial and neuronal cells has not previously been studied with probes of same size, shape and material. This is crucial since, for example, it is known that small implants tend to evoke less reactions than large implants [11] and different types of materials and shapes may cause different tissue responses [22]. The decrease in reactive astrocytosis, indicated by GFAP-density, after aligning the implants direction of flexibility to the main direction of movement of the brain (i.e. the coronal axis; flex mode) supports the hypothesis that an implant able to move with the tissue will evoke a smaller response. Hence, the astrocytic scar tissue appears to be dependent on the shear stress between implant and tissue resulting from rigid probes tethered to the skull. However, we found no significant impact on microglia recruitment of probe orientation, indicating that the difference in shear stress caused by the two probe orientations used has not a marked effect on microglial activation. Moreover, the loss of neurons in the inner ROI was about the same for the two implantation orientations. Hence, although we cannot entirely rule out a difference in the local tissue damage caused by the two implantation modes, this does not seem to be a major confounding factor.

### Effects of gelatin embedding on glial cells

In this study, we found that gelatin embedding significantly reduced the microglia response while having no obvious effect on astrocyte proliferation. The significant effect on microglia response support previous findings [18] that gelatin embedding reduce microglial responses to the implant. On implantation, gelatin quickly becomes slippery thereby reducing friction with tissue. This may reduce the acute tissue damage on implantation, which is a trigger for microglia activation. The lack of effect of gelatin on long-term astrocyte proliferation may be related to that the effect of gelatin was analyzed using probes implanted in flex mode which already exhibit significantly reduced astrocyte proliferation in the non-embedded group (as discussed above). As gelatin is quickly dissolved in the tissue, the failure of gelatin-embedding to reduce the astrocytosis further may suggest that continuous shear stress caused by a rigid implant is of key importance for astrocyte proliferation [20,23,24,25].

### Effects on neuronal density and morphology

Importantly, no obvious loss of neurons in the inner ROIs for the gelatin embedded probes were found, whereas there was a reduced survival of neurons in the inner ROIs of the non-embedded probes, as previously reported [19]. These results support previous findings suggesting that gelatin embedding is beneficial for neuronal survival [26,27]. It should also be noticed that neurons in the inner ROI appeared less deformed when implants were allowed to flex in the principal direction of movement within the brain parenchyma (Fig. 3A). The absence of a reduction in number of neurons and less morphological changes observed indicate that these neurons are much less affected by the presence of the implanted probe as compared to state of art electrode implants [28].

In the outer ROIs, no differences between probes implanted in either flexible or rigid modes were found. This is expected in light of the size-dependent decrease in tissue response along with decreasing probe size observed in previous studies. Indeed, probes a few tenths of micrometers thick provoke small or no observable immune responses outside the 50 μm ROI even with stainless steel probes considerably bulkier than the probe used in this study.[11]

## Conclusions

This work is part of a series of studies aiming at elucidating which factors are important for glial scarring and subsequent displacement or loss of neurons adjacent to implanted neural interfaces. To simplify the analysis, we used probes of same material, size and shape but with substantial difference in flexibility with respect to the directions of the principal movements of the brain relative to the skull. We here provide evidence for that astrocytic proliferation depends, at least partly, on whether implanted probes are able to follow tissue movements. The microglial responses to such probes can be further improved by embedding them in gelatin. Notably, gelatin embedded flexible probes also appear to be beneficial for neuronal survival in the inner ROI.

## Supporting Information

S1 File Contains the raw data and statistical evaluations. Sections in the data base are arranged in a corresponding way to the presentation of data in the Result section, i.e. GFAP, ED1, NeiN and DAPI. The file is oranized using GraphPad Prism 5.02 software (GraphPad Software Inc., USA). The software is available at www.**graphpad**.com/scientific-software/**prism**.
(PZF)
